# Challenges and Future Directions in the Management of Tumor Mutational Burden-High (TMB-H) Advanced Solid Malignancies

**DOI:** 10.3390/cancers15245841

**Published:** 2023-12-14

**Authors:** Jibran Ahmed, Biswajit Das, Sarah Shin, Alice Chen

**Affiliations:** 1 Developmental Therapeutics Clinic (DTC), National Cancer Institute (NCI), National Institute of Health (NIH), Bethesda, MD 20892, USA; 2 Molecular Characterization Laboratory, Frederick National Laboratory for Cancer Research, Frederick, MD 21702, USA

**Keywords:** TMB, tumor mutational burden, ICI, immune checkpoint inhibitor, biomarker

## Abstract

**Simple Summary:**

When employing a high tumor mutational burden (TMB) as a predictive biomarker for immune checkpoint inhibitor (ICI) response, one faces various challenges. It is important to understand TMB in the context of the tumor microenvironment and genomic features. This review provides an update on these challenges and offers insights into improvement strategies for the future, aiming to enhance the predictive accuracy of this biomarker for clinical use.

**Abstract:**

A standardized assessment of Tumor Mutational Burden (TMB) poses challenges across diverse tumor histologies, treatment modalities, and testing platforms, requiring careful consideration to ensure consistency and reproducibility. Despite clinical trials demonstrating favorable responses to immune checkpoint inhibitors (ICIs), not all patients with elevated TMB exhibit benefits, and certain tumors with a normal TMB may respond to ICIs. Therefore, a comprehensive understanding of the intricate interplay between TMB and the tumor microenvironment, as well as genomic features, is crucial to refine its predictive value. Bioinformatics advancements hold potential to improve the precision and cost-effectiveness of TMB assessments, addressing existing challenges. Similarly, integrating TMB with other biomarkers and employing comprehensive, multiomics approaches could further enhance its predictive value. Ongoing collaborative endeavors in research, standardization, and clinical validation are pivotal in harnessing the full potential of TMB as a biomarker in the clinic settings.

## 1. Background

Tumor Mutational Burden (TMB) has emerged as a promising genomic biomarker of response to immune checkpoint inhibitors that was pioneered by Rizvi and Chan [1]. TMB is defined as the total number of mutations within a tumor genome (mutations per megabase of genome or mut/Mb) and serves as a measure of the potential neoantigen load that may elicit an anti-tumor immune response. This is based upon the type of mutations, a frequency of threshold in the context of clinical activity, the type of cancer cohort, and the sequencing methods used (whole-exome sequencing (WES), whole-genome sequencing (WGS), targeted panel-based next-generation sequencing (NGS)) [2].

TMB molecular signatures are varied, and they arise from a combination of both exogenous and endogenous factors, which represent environmental factors that affect mutagenesis rates and mutations due to random errors in deoxyribonucleic acid (DNA) replication, respectively [3]. This molecular signature has been shown to be predictive of response to immune checkpoint inhibitors (ICIs) in several clinical trial settings. Similarly, hypermutated tumors vary in their causative factors, for example, UV (ultraviolet) light for skin cancer and smoking in non-small cell lung cancer (NSCLC), as well as somatic mutations. In addition, there is a huge variability in mutational load across cancers, with melanoma and NSCLC frequently showing a high TMB. Thus, defining a median range across several cancer types is not practical and other approaches might serve as useful predictive biomarkers of response [4]. Despite these adjustments, cancers such as renal cell cancer (RCC) may not have a high TMB but respond well to ICI.

High TMB is common among several cancers, with an incidence of TMB ≥ 10 mut/Mb of 14.7% based on the TSO500 (TruSight Oncology 500, Illumina, San Diego, CA, USA) panel assay in one study and a pooled overall prevalence of 14% on panel-based or WES assays in solid tumors [5,6]. Among less common tumors, including but not limited to biliary tract cancers, small cell lung cancer, and mesothelioma, the prevalence of TMB-H (≥10 mut/Mb on FoundationOne®CDx (F1CDx) panels) was noted to be 12.8% [7].

Studies have suggested that a tumor with high TMB enhances the probability of the formation of neoantigens and the potential for recognition by the immune system, thereby making these tumors sensitive to immune checkpoint blockade [8,9].

In the last few years, TMB has gained wide acceptance as a predictive biomarker of ICI therapy response. The FDA (U.S. Food and Drug Administration, Silver Spring, MD, USA) has cleared multiple tests as either companion diagnostic tests or class II devices [10]. Despite an interest in the prospective clinical utility of TMB, significant challenges remain in TMB testing and standardizing methodology, preventing its use as a robust predictive biomarker for ICI therapy. The molecular complexity and heterogeneity inherent in cancer form the foundation for the imprecision observed in molecular-based biomarkers. Tumor heterogeneity, for example, implies variation in genomic profiles, treatment sensitivity, and resistance mechanisms within a tumor and among their sites of metastasis [11]. This review examines these challenges and variations associated with testing to integrate TMB as a biomarker within the clinical context while also addressing its prospective use. The proposed future directions for this biomarker could have implications for other molecular diagnostics and therapy.

## 2. Challenges of Using TMB as Biomarker of Response to ICI

Whole-exome sequencing (WES) encompasses 32 Mb of the coding region, the entire set of 22,000 genes, which constitutes roughly 1% of the genome. WES, the gold standard for TMB calculation, measures the number of somatic alterations, whereas the FDA-authorized surrogate panel assays including the Memorial Sloan-Kettering-Integrated Mutation Profiling of Actionable Cancer Targets or MSK-IMPACT (468 genes) and F1CDx assay (324 genes) measure the density of somatic alterations, including target footprints of approximately 1.14 Mb and 0.8 Mb of the coding regions, respectively [12,13]. Numerous studies have shown a correlation between WES TMB and panel-based TMB (pTMB); nevertheless, the overestimation of TMB by panel assays and other differences exist [4,14].

Keynote-158, the pivotal trial leading to the tissue-agnostic approval of pembrolizumab, evaluated tissue TMB (tTMB) in FFPE (Formalin-Fixed Paraffin-Embedded) tumor samples through the utilization of the F1CDx assay with a predefined criterion for classifying high tTMB as the presence of a minimum of 10 mut/Mb. The trial showed an objective response rate (ORR) of 29% in the tTMB-high versus 6% in the non-tTMB-high groups when treated with pembrolizumab at 200 mg IV (intravenously) every 3 weeks until unacceptable toxicity or disease progression [15].

The overall survival (OS) and progression-free survival (PFS) were secondary outcomes in the trial, and a mOS (median OS) benefit was not seen (11.5 months in t-TMB-high versus 12.8 months in non-tTMB-high group). The trial was certainly limited in the types of cancers (it included nine different cancer types) and patient numbers, including cancers with relative resistance to immunotherapy. Fourteen percent of patients assessed for efficacy in the TMB-high group also had Microsatellite Instability-High (MSI-H); however, after excluding patients with missing or MSI-H status, the ORR was 28%. In addition, 67% were positive for PD-L1 (Programmed death-ligand 1) CPS (combined positive score) ≥1%, although an association of efficacy was not seen among patients with tTMB scores and PD-L1 CPS positive scores. Subsequent observations in a single institution study indicated that the OS benefit is lost in CRC (colorectal cancer) patients when categorized based upon the presence of pathogenic mutations in *POLE* (polymerase ε) or *POLD1* (polymerase δ1) (hazard ratio 1.17) [16]. These mutations are frequently present in a hypermutated cancer phenotype [17].

**Figure 1:**
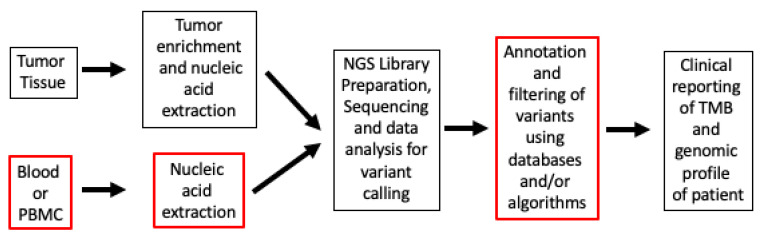
TMB workflow from tissue specimens to clinical report. The boxes with red outlines are steps that are substantially different in labs and workflows. Abbreviations: NGS, next-generation sequencing; PBMC, peripheral blood mononuclear cells; TMB, Tumor Mutational Burden.

A retrospective analysis of several keynote trials (Keynote-001, Keynote-002, Keynote-010, Keynote-012, Keynote-028, Keynote-045, Keynote-055, Keynote-059, Keynote-061, Keynote-086, Keynote-100) demonstrated improvement in ORR among patients treated with ICI, with tTMB ≥ 175 mutations/exome compared to TMB < 175 mutations/exome (31.4% vs. 9.5%, respectively). Additionally, an analysis of patients in Keynote-010, Keynote-045, and Keynote-061 showed improvements in PFS and OS compared to chemotherapy [18]. A more recent retrospective analysis also showed that patients in Keynote-042 derived PFS and OS benefits if their tTMB was ≥ 175 mutations/exome compared to tTMB < 175 mutations/exome [19]. Multiple other systematic reviews and metanalyses have indicated a strong association between high TMB and enhanced efficacy, with several studies also highlighting improved survival outcomes across cancers and in specific cancer types [20,21,22]. When it comes to evaluating TMB, inherent differences exist in testing workflow methods (Figure 1).

While WES and MSK-IMPACT use similar filtering strategies of using non-synonymous mutations for calculating TMB, tumor-only sequencing panels (like F1CDx) also include short indels in introns and synonymous mutations in the panel testing for TMB. And, considering that neoantigens are formed by non-synonymous mutations only, the synonymous mutations are included in TMB calculation to reduce sampling noise and increase the robustness of TMB scoring methods [23]. In some cases, the use of synonymous mutations in conjunction with a statistical framework substantially increased the concordance of TMB values with a WES-based reference method [24]. However, the F1CDx test result reports TMB-H when it is found to be ≥20 mut/Mb, and intermediate and for select tumors when TMB is 6–19 mut/Mb; TMB-low is listed under the VUS (Variants of Uncertain Significance) section of the report.

Table 1 outlines some of the challenges linked to TMB assessment and proposes potential solutions.

Additional differences in the panel content among the commercial panel assays may give rise to inter-assay variations. For example, in focused panels, there may be higher detection rates of pathogenic driver mutations compared to the background mutation rate in the tumor. This may lead to higher levels of variability at low TMB values [26]. Therefore, it may be important to calibrate the thresholds for each panel in addition to generating an adjusted score (e.g., “mutational load”) by taking into account the variability within tumor types [31]. This may help in better interpretation of the TMB scores. Many of these panel-adjusted scores used in determining TMB also show a good correlation to TMB scores generated from WES and WGS [32], whereas the TMB calculation from WES and WGS seems to be highly concordant, although the data analysis pipeline for WES is less intensive and less costly [32,33].

Several factors impact the TMB calculation among different panel tests. The first of these is the size of the panel variation, such as 0.8 Mb in F1CDx versus 1.94 Mb for the TSO500 panel. Studies have shown that a smaller panel size may produce more misclassifications of TMB calculation compared to a larger panel size. This includes variation in thresholds among smaller compared to larger panel sizes. In particular, a smaller panel size is less precise in distinguishing between hypermutated from non-hypermutated cancers [34]. Smaller panel sizes also tend to overestimate TMB values whereas panel tests may overestimate TMB compared to WES [35]. Budczies, A. et al. reported that the coefficient of variation (CV) of TMB derived from panel sequencing data decreased at a rate inversely proportional with the square root of the panel size and the square root of the TMB level [36]. Additionally, panel sizes of >667 Kb (kilobase of DNA) have been shown to be adequate to maintain standardization [27].

Inter- and intra-tumor heterogeneity can produce imprecise TMB measurement [37]. TMB from metastatic sites could be higher than that from primary sites due to varying clonal heterogeneity, and yet, this disparity may not necessarily influence the survival benefit derived from ICI therapy [38]. Moreover, the presence of organ-specific niches such as bone metastasis may host resistance mechanisms that result in diminished clinical responses to ICI therapy [39]. The locations of sequenced regions and types of mutations included also vary among different panel-based assays that are used to calculate TMB. In addition, tumors are known to have clonal evolution during the course of treatment, which may preferentially impact the non-neoantigenic pathways, thus making TMB interpretation variable across the tumor course [40].

Tumor-targeting cytotoxic T lymphocyte variation in the cellular neighborhoods (CNs) is dynamic and impacts response to ICI therapy, with HLA-1 expression downregulation predicting poor outcomes with ICI in colorectal cancer patients [41].

Another challenge lies in the influence of tumor purity, such as that related to tissue sampling, where the low tumor cellularity could result in falsely low TMB measurement [42]. The exclusion of germline alterations also varies across different panels. For example, WES excludes germline variants using patient-matched samples and MSK-IMPACT uses patient-matched blood samples. In contrast, the F1CDx panel eliminates germline variants by using in silico bioinformatics algorithms. Methods of germline variant subtractions may lead to significant discordance for TMB calculation for panel tests [28]. Alternatively, germline molecular signatures associated with high TMB tumors may respond better in terms of survival and a consideration for standardizing these signatures in important [43]. Continued improvement in data annotation filtering processes is needed.

The composition of the panel test, with the variable selection of genomic alterations, can also produce variability in TMB calculation [36]. After accounting for artefacts and germline variation, a panel test comparison shows a good correlation with the inclusion of synonymous and coding non-synonymous alterations [44].

Overestimation in TMB can also occur by including mutations with VAF (variant allele frequency) only above a certain threshold [45]. Hence, the specific context of the underlying mutation (synonymous, non-synonymous, or Indels) and whether it occurs in the coding or non-coding regions might contribute to minor variation in TMB calculation across different panels. Moreover, mutation calling tools, especially when identifying low-frequency alterations, may necessitate a deeper sequencing depth, which could result in potential omissions [46]. While these tools can also help in omitting clonal hematopoiesis and resistance mutations, with the increasing comprehension of novel primary and secondary resistance mechanisms, we must account for these in TMB estimation [47].

## 3. Factors in Tumor Microenvironment (TME), TMB, and Response to ICI

The tumor microenvironment (TME) constitutes a diverse ecosystem, and to harness the entire TME for improved immunotherapies, it is crucial to recognize that multiple immune subsets play a role in shaping the variability in immune response [48,49]. Thus, these anti-tumor immune responses are complex and involve several factors driving the cancer immunity cycle that promote or suppress anti-tumor immunity [50]. Simply relying on a single biomarker such as TMB to explain the response to ICIs may not capture the intricate interplay of sensitivity and resistance mechanisms underlying the use of these therapies [51].

It is also known that beyond tumor histology, there are several other mechanisms that can impact response to ICIs, such as cellular signaling, checkpoint signaling pathways, immune cell activity, variability in HLA expression and TCR repertoire, the gut microbiome, and oncogenic signaling pathways indirectly associated with response to ICIs [52,53].

Exploration in this field is an active area of clinical and translational research. This involves combining PD-1/PD-L1 (anti-programmed death receptor-1/anti-programmed death ligand-1) inhibitors with other immune-modulating or targeted agents, depending on the stromal environment of the tumor, for example in hot versus cold tumor microenvironments. Several combinatorial checkpoint agonists/antagonists, as well as targeted therapies combined with immunotherapies (such as KRAS (kirsten rat sarcoma virus) inhibitors inhibitors with pembrolizumab), are being studied in clinical trials [54]. Such endeavors will also be tested within the clinical trial framework of the iMATCH master protocol sponsored by the NCI (National Cancer Institute) [55].

A higher HLA class II expression has been shown to be associated with positive tumor responses through correlative analysis in CheckMate 064 and CheckMate 069 [56]. Similarly, heterozygosity in HLA-1 is associated with better response to ICIs [57].

Immunologically cold tumors have lower response rates to ICIs. However, TMB-H is not always correlated with CD8+ tumor-infiltrating T-cells, since a portion of CD8+ T-cells are bystanders and recognize antigens unrelated to tumors [58,59]. Furthermore, it has been shown that the quantity of neoantigens and the extent of mutational load are comparable in melanomas characterized by T-cell inflammation versus those lacking T-cell inflammation [60].

A post hoc pan-cancer analysis using MSK-IMPACT for TMB scores showed that the OS among patients treated with ICIs was associated with sets of genomic alterations in TMB-low versus TMB-high tumors. In particular, hypermutation (TMB ≥ 100 mut/mb) is associated with certain genomic signatures including *POLE*/*POLD1*, dMMR (deficient mismatch repair), the activation of AID/APOBEC (activation-induced cytidine deaminase/apolipoprotein B mRNA (messenger ribonucleic acid) enzyme catalytic polypeptide-like), and the three clock-like mutational processes (SBS1, SBS5) [26].

Somatic mutations have the potential to generate neoantigens, and the resulting cancer-specific genomic signatures can vary across cancer types [61].

In a study by Negrao M. V. et al., oncogene-dependent lung cancers (including alterations in *EGFR* (epidermal growth factor receptor), *KRAS* (Kirsten rat sarcoma virus), *BRAFV600E* (v-raf murine sarcoma viral oncogene homolog B1), *HER2* (human epidermal growth factor receptor 2), *MET* exon 14 skipping mutations, and *RET*, *ROS1*, and *ALK* (Anaplastic lymphoma kinase) gene fusions/rearrangements) exhibit variable TMB expression and outcomes with ICIs, generally showing a low TMB (<10 mut/Mb) among these common oncogenic groups [62]. Alterations including *KRAS*, *EGFR*, *STK11* (Serine/Threonine Kinase 11), *KEAP1* (Kelch-like ECH-associated protein 1), and *MDM2* (Mouse double minute 2 homolog), among others, may impact immunotherapy sensitivity and outcomes in NSCLC [63].

The impact of driver genes on TMB is noteworthy in NSCLC, wherein high TMB may be linked to reduced survival in *EGFR* mutated cancers [64]. Specific mutations, including *CDH1* (cadherin-1), *RAD50*, and *MSH2* (muts Homolog 2), have been associated with high TMB in head and neck squamous cell cancers [65]. Certain mutations linked to responses to ICIs have been observed [66]. For example, in melanoma, mutations in *JAK1*/*JAK2* (Janus Kinase 1/2) and *B2M* (beta-2-microglobulin) genes associated with acquired resistance to ICIs, and *STK11*/*LKB1* mutations in *KRAS* mutated lung adenocarcinoma, have been documented [67,68]. The impacts of specific mutations and mutation signaling pathways, and their effects on immunogenicity and link to TMB, require extensive elucidation [1,69,70]. Additional information on other factors influencing TMB is covered in the section on future directions.

## 4. Efficacy and Real-World Data on TMB Testing

Several clinical trials have demonstrated the clinical utility of TMB as a predictive biomarker of response to single-agent and combination immunotherapy [71]. In general, there is substantial real-world evidence indicating a response and enhanced survival in cases of TMB-H tumors, with varying definitions, but primarily in TMB thresholds ≥ 10 mut/Mb. The analysis of large clinico-genomic databases to assess real-world OS analyses of TMB has shown the benefit of high TMB across 24 cancer types compared to a low TMB [72]. Another retrospective analysis of patients with MSI-H and/or TMB-H (≥20 mut/Mb on F1CDx) among 27 different cancer types showed better PFS outcomes with immunotherapy in patients who had previously received chemotherapy [73]. A study in NSCLC demonstrated an increase in real-world OS as TMB scores increased from <10 to 10–19 and ≥20 mut/Mb (10.1, 11.8, and 26.9 months, respectively) [74].

## 5. Future Directions

Precision in patient selection remains a pivotal consideration in selecting TMB as a predictive biomarker in cancer patients. Simply raising the threshold for TMB may lead to a smaller intention-to-treat population. However, based on the treatment context, the adoption of cancer-specific thresholds may impact the clinical outcomes [75]. Nevertheless, it is crucial to find the right equilibrium when adjusting for a cancer-specific TMB threshold. Setting the threshold too high might result in the exclusion of patients who could potentially benefit from immunotherapy.

This entails achieving standardization in TMB assessment at each step, including sample collection, DNA extraction and processing, sequencing techniques, bioinformatic processing, and reporting on TMB scores (Table 1).

Standardization initiatives could encompass WES panels, covering both synonymous and non-synonymous mutations for panel tests; the inclusion of driver alterations, germline mutations, and genomic signatures that may impact responses to ICIs; the subtraction of germline alterations with concurrent blood-based testing or bioinformatics pipelines; and the subsequent development of standardized pipelines, quality control (QC) metrics, and annotation tools that ensure the consistent identification and interpretation of TMB across labs and research projects.

### 5.1. Standardization Efforts of TMB Reporting: Tumor Mutational Burden (TMB) Harmonization Project

The TMB harmonization project phase I (in silico analysis) and phase II (empirical analysis), comprising a joint initiative involving the Quality Assessment Service for Pathology (QUIP) and Friends of Cancer Research, have resulted in an endeavor to standardize TMB reporting.

In the first phase, panel TMB, calculated from Insilco WES data from The Cancer Genome Atlas (TCGA), was compared with a collectively established reference, WES TMB, using bioinformatics pipelines, and the project members recognized tumor and panel variability among these panels [76]. The empirical analysis of cell-line and clinical samples in phase II of this project established the importance of panel coverage, germline mutation calling, and calibrating panel assays [27]. Continued clinical assessment in retrospective analyses is required for TMB validation.

### 5.2. Integration with Other Biomarkers and Clinical and Genomic Data

Potential biomarkers for ICI therapy include tumor-intrinsic biomarkers such as TMB, neoantigens, PD-L1 expression, and MSI status; immune-specific biomarkers such as T-cells, T_effector_/T_regulatory_ ratio, tertiary lymphoid structures (TLSs), γ-IFN (gamma interferon), and B cell signatures; and combinatorial biomarkers such as TMB and PD-L1 [77,78].

MSI-H and TMB both share genomic instability mechanisms and increased immunogenicity [79]. Most of the MSI-H tumors are also TMB-high. However, other aberrant biological processes in tumors may also result in high mutational rates (e.g., UV exposure, mutations in *POLE* and *POLD1* genes) [80,81]. And, although they stem from different mechanisms (MMR deficiency for MSI-H and general genomic instability for high TMB), the result is a higher tumor mutation load in both cases. Thus, it is frequently noted that MSI-H tumors such as colorectal and endometrial cancers frequently have TMB-high [23]. This synergistic effect has been studied in terms of response to ICIs. However, this correlation seems to be tumor-specific and may not be present across all solid tumors [82]. Given that the combined MSI-H and TMB-H status is not universal among all cancers, research has indicated that individuals with MSS (microsatellite stable) tumors and high TMB levels can derive clinical benefit from ICIs, and, in fact, the discordance between the two biomarkers may be responsible for ICI resistance [83,84].

Combining and incorporating other biomarkers such as PD-L1 may increase the predictive and prognostic capabilities in decision making for ICI therapy [85]. However, the TMB and PD-L1 may not be well correlated across tumor types [86,87]. An exploratory analysis of patients who received nivolumab in checkmate 026 showed an improvement in ORR (75%) among patients with PD-L1 ≥ 50% and TMB-H [88]. On the other hand, in the checkmate 227 trial, the response in TMB-H and PD-L1 ≥ 1% patients treated with nivolumab plus ipilimumab (versus chemotherapy) was comparable to that observed in patients with low PD-L1 and low TMB [89]. Whereas, in CheckMate 568 (Nivolumab plus Ipilimumab in advanced NSCLC), the higher ORR was noted for patients with TMB-high tumors (≥10 mu/Mb using the F1CDx platform) regardless of PD-L1 status [90].

Other markers of sensitivity to ICIs such as CD-8+ T-cell abundance, inflammatory TME, and T-scores may inform a composite biomarker for future use. However, in a retrospective analysis, McGrail D.J. et al. showed that in cancer patients with no positive correlation between neoantigen load (a TMB surrogate) and CD8+ T-cell levels, a high TMB was not a predictive biomarker of ICI therapy. High TMB also did not predict response in cancers such as brain, breast, and prostate cancers [58]. Thus, further studies are needed, perhaps at variable TMB thresholds, to establish the true usefulness of TMB among these less immunogenic and other less common tumor types. Collinearity assessment and multifactor analysis are needed to assess the impact of TME and TMB scores on ICI outcomes.

Cristescu R. et al. studied the combined relationship between TMB and T-cell-inflamed genomic expression profile (GEP) scores and showed that among patients treated with pembrolizumab in certain Keynote trials (Keynote-012 B1, Keynote-012/028, Keynote-012 B1 + B2, Keynote-001, and Keynote-006), GEP^hi^ scores (top pan-cancer tertile value) and TMB-high scores calculated from WES (TMB^hi^ assessed using Youden Index cut-offs) were correlated with an ORR and PFS benefit among pan-tumor, head and neck, and melanoma patient cohorts [91]. However, the overall number of correlations, in the pan-cancer group, of GEP and TMB scores were low.

Tertiary lymphoid structures (TLSs) are now recognized as important mediators of anti-tumor immunity and lymphocyte response [92]. Thus, the TLS has emerged as a potential biomarker due to its association with survival with ICI therapy [93]. A clear correlation between TLS and TMB has not been well established when comparing ICI responses [94,95]. In a more recent study, the presence of mature tertiary lymphoid structures (mTLS) on solid tumor samples was demonstrated to be significantly associated with high bTMB (blood-based TMB) compared to those with the absence of mTLS (16.7% vs. 8.5%, *p* = 0.0036) [96].

The neutrophil lymphocyte ratio (NLR) and the levels of peripheral monocytes and neutrophils represent additional biomarkers that may predict response to ICIs, and the pre-treatment NLR could serve as another candidate for a potential composite biomarker when combined with TMB [97,98]. In this context, a low NLR with a high TMB ratio may indicate a more favorable response to ICI therapy [99].

HRD (homologous recombination deficiency) alterations and genomic instability may have substantial associations with high TMB [100]. Heterozygous HLA (Human Leukocyte Antigen) types have been shown to have a better response to ICIs, and studies have shown that HLA-LOH (HLA-Loss of Heterozygosity) which is linked to immune escape in lung cancer may be associated with a high TMB [101,102]. Thus, the idea that an HLA-corrected TMB may be associated with a survival advantage in NSCLC patients has been proposed [100]. An analysis of genomic scars using the HRDsum score, calculated by combining LOH, telomeric–allelic imbalance (TAI), and large-scale state transitions (LSTs), showed a positive correlation with TMB scores (Spearman’s rho of 0.6, 0.52, 0.43 and 0.43 for pancreatic adenocarcinoma, breast cancer, ovarian cancer and prostate adenocarcinoma respectively) [103].

Furthermore, the baseline clonality and diversity of T-cell receptor (TCR) profiles have demonstrated potential predictiveness for response to ICI treatment [104,105]. For instance, in the context of breast cancer patients undergoing neoadjuvant chemotherapy, there appears to be an association between response outcomes and high TMB and TCR clonality [106].

Metabolites produced by the gut microbiota, such as short-chain fatty acids (SCFAs), have the potential to modulate ICI therapeutic effects and toxicity [107,108]. Importantly, the gut microbiome has been shown to alter the response to ICIs, with microbiome composition, diversity, load, and dysbiosis impacting this response [109]. More studies are needed to identify the role of microbial diversity in modifying the TME with the potential increase in mutational load. The identification of specific microbial signatures associated with high TMB and other ICI biomarkers is also needed.

The commercial availability of WES is still limited, primarily within research settings; however, there exists a potential for increased accessibility in the future [110]. Although it currently may not be cost-effective for clinical application, the use of WES could become more common, paving the way for the broader incorporation of additional markers associated with TMB and the response to ICIs [111].

With the emergence of several new sequencing panels and ongoing enhancements to existing ones, alternative methods of comparing panel assays for TMB calculation, such as angular distance, may be employed alongside the conventional R-squared value obtained through linear regression and Spearman’s rank correlation [14].

In the future, it will be necessary to account for the influence of the tumor landscape on TMB in specific cancer types and with a further consideration for assessing the neoantigen landscape of these tumors since TMB represents a surrogate to neoantigen load in cancer cells [112]. In one study on the clinical outcome of pembrolizumab versus chemotherapy in lung cancer patients, it was observed that the treatment outcomes appeared to be comparable irrespective of the presence of *STK11*, *KEAP1*, or *KRAS* mutations [113]. Meanwhile, the impact of individual mutations and signatures on TMB scores may vary [114]. As an example, *ARID1A* (AT-rich interactive domain-containing protein 1A) mutation is associated with a high TMB and MSI-H tumors, as well as improvement in PFS after ICI therapy [115,116]. Additional contributors in the tumor microenvironment include chemokines, and studies suggest that specific chemokine signatures could serve other predictive factors for response to ICIs [117]. A correlation between chemokine signatures and TMB and the combined impact on outcomes needs to be elucidated [118]. The upregulation of the RNA expression of chemokine *CXCL13* (CXC-chemokine ligand 13) was correlated with a better response to ICI therapy in lung adenocarcinoma patients; however, this overexpression was not significantly associated with TMB (*p* = 0.054) [119].

There is a necessity for the adoption of a multiomic strategy to discover markers that can predict response to immunotherapy. Using multiomic analysis, Li Gao et al., showed that certain alterations such as *ROS1* (c-ros oncogene 1), *SPEN* (spen family transcription repressor), and *PTPRT* (Protein Tyrosine Phosphatase Receptor Type T) were predictive of a response to immunotherapy across several cancer types. The variation in response was also significant for various clinical and demographic factors [100].

### 5.3. TMB in Blood

Non-invasive blood-based TMB (bTMB) has emerged as a promising tool to assess TMB in the clinic for ICI treatment [120]. The robustness of bTMB measurement can be variable and dependent on the technical features of ctDNA assay including factors such as the sensitivity of the variant detection, precision of bTMB calling, and biological factors such as tumor fraction in plasma cfDNA (cell-free DNA) and the heterogeneity present in the ctDNA (circulating tumor DNA). Due to these issues, concordance levels between bTMB and tTMB can be low [121]. These challenges can adversely affect the interpretation of bTMB results and the value of bTMB as a biomarker of response to ICIs. Examples of clinical trials validating bTMB are presented in Table 2.

In addition to its increasing use, there is also accumulating real-world evidence of the predictive efficacy of bTMB in NSCLC [130]. This concordance between bTMB and tTMB remains evident when comparing different NGS assays in the context of NSCLC treated with ICIs [131]. Paired testing using tumor and blood TMB assessment may offer a benefit to reduce variations in tumor heterogeneity [132]. The clinical use of bTMB on ctDNA requires further standardization for use as a dynamic biomarker. This becomes particularly crucial when assessing cancers longitudinally, especially in cases where obtaining repeat tumor biopsies poses challenges. Harmonization endeavors will be needed in the process of genomic profiling and predicting the response to ICI through the evaluation of biomarkers assessed by circulating tumor cells (CTC), ctDNA, and extracellular vehicles (EVs).

### 5.4. TMB as a Prognostic Biomarker

A retrospective analysis that was conducted to evaluate the prognostic value of TMB-H (defined as ≥10 mut/mb) in patients who had not received immunotherapy showed an overall prevalence of TMB-H of 12.8% and failed to demonstrate a OS benefit in patients with TMB-H compared to those without TMB-H (HR 0.94) [7]. However, the impact of high TMB on survival may have varying prognostic effects depending on tumor subtypes [133]. Another retrospective study showed that a high TMB may be prognostic for longer survival in patients not previously treated with ICIs [134].

There is a paucity of data on the role of TMB in the neoadjuvant setting. One example is the Keynote-816 trial in resectable NSCLC treated with nivolumab compared to nivolumab plus chemotherapy [135]. In this study, the estimation of the 95% confidence interval for event-free survival of both TMB thresholds of < or ≥12.3 mut/Mb crossed the line of no effect, suggesting no influence of TMB in this setting.

### 5.5. Expressed TMB (eTMB)

Recently, there has been a notable increase in developing omics, and RNA-seq (RNA sequencing) provides an opportunity to study expressed somatic nucleotides to estimate a biologically relevant computation of TMB in comparison to TMB estimated using WES and panel testing [136,137]. In addition to expression data, the DNA sequencing methods do not cover all gene fusions and alternative splice site variants that impact the TMB calculation. Specialized bioinformatic pipelines for variant calling and comparison to WES are emerging with the specific goal of unraveling the relationship between transcribed and expressed TMB and TMB estimated using genomic methods [138]. More data are necessary to validate this technique and prospectively evaluate its impact on the outcomes of patients undergoing ICI treatment.

### 5.6. Additional Considerations for Future

Several other TMB measurement methods have been proposed and could be considered part of the harmonization efforts for TMB. For example, a background mutation rate (BMR) modeling method to calculate an ecTMB (estimation and classification of TMB) has been shown to produce consistency across panels and better prediction of TMB in panel assays [24]. Novel methods such as RNA sequencing can account for several inconsistencies in currently used methods by providing consideration for the tumor heterogeneity, driver mutations, and signatures that impact TMB calculations [138].

While the universal TMB cut-off of 10 mut/Mb is deemed optimal across several different cancer types, considering the substantial variation in TMB levels among different cancer types, one might employ tumor-specific, non-universal thresholds tailored to specific cancers, or even using higher percentiles (e.g., the top 20% within each histology), medians of the interquartile range, the z-score conversion of TMB, or multiple-tier thresholds (dose-dependent effect), for TMB calculation and reporting [4,75,139,140,141]. In one study, it was demonstrated that the clinical advantage of using a TMB ≥ 16 mut/Mb was amplified when considered in conjunction with microsatellite status and PD-L1 expression profile [5]. Raising the TMB threshold (for example, to the 90th percentile) may decrease the intention to treat population numbers; however, it may also be associated with greater improvement in survival among various cancers [142].

Tumor indel burden, which includes small frameshift insertions and deletions, has been assessed in comparison to TMB across several cancer types. For example, in a study by Wu, W. et al., the authors noted that TMB was prognostic, and remarkably, indel burden had a different impact on OS in some cancers when compared to TMB [133]. Using tumor variant burden (TVB) and neoepitopes by combining DNA and RNA sequencing data did predict for a response immunotherapy [143]. A meticulous determination of histology-specific TMB cut-off is also needed for rare tumors, especially those not included in clinical trials such as Keynote-158 and other trials employing TMB as a predictive biomarker.

There is an ample amount of data supporting the dynamic nature of immune response that undergoes continuous evolution, with the emergence of compensatory immune-inhibitory pathways following treatment with ICI therapy [144]. Several checkpoint agonists/antagonists are in clinical development alone or in combination with anti-PD-1/anti-PD-L1 inhibitors [145]. These include antibodies targeting LAG-3 (Lymphocyte Activation Gene 3), TIM-3 (T-cell immunoglobulin and mucin domain-containing protein 3), VISTA (V-domain Ig Suppressor of T-cell Activation), and TIGIT (T-cell immunoglobulin and ITIM domain) among several other targets [146]. And, although TMB may be an established predictive biomarker of response in patients treated with anti-PD-1/anti-PD-L1 therapy alone or in combination with anti-CTLA-4 therapy, the use of TMB for predicting response to ICIs in combination with other checkpoint therapies needs to be elucidated [147,148].

The evolution of TME composition in advanced cancers is a proposed mechanism of resistance to ICIs, particularly in later lines of treatment [47,65,149]. In this context, it is necessary to further elucidate the role of TMB as a dynamic biomarker across different stages and metastatic sites of cancer. This could also imply that TMB thresholds may differ depending on the clinical stage and extent of prior therapy in different cancers. Studies have indicated predictability in anticipating responses to ICIs in neoadjuvant or early-stage scenarios across various cancer types, including but not limited to NSCLC, melanoma, breast cancer, and HNSCC [106,150,151,152]. A study by Niknafs, N. and colleagues has shown an association between underlying molecular alterations and the emergence of a persistent TMB that is linked to a favorable response to ICIs [153].

AI and machine learning have surfaced as novel tools for large-data analysis and interpretation, potentially offering valuable contributions to TMB assessment when dealing with large data sets. These applications encompass the potential use of radiomics and histopathologic imaging to improve TMB prediction, as well as integrated multiomics to form prediction models to enhance the performance of TMB assessment [154,155,156].

Another aspect in TMB assessment is the consideration of the impact of race and ethnicity on germline variants since the databases are biased toward, for example, white populations, and ethnicity-related aspects require careful analysis in this context [157,158]. The removal of germline variants for TMB calculation is important considering ethnic groups may not be adequately represented in SNP (single nucleotide polymorphism) databases. A study by Hsiehchen D. et al. noted differences in distribution and response to ICIs based on race among a TMB-H population. High TMB was less often seen in Asian and Black populations compared to white populations, and although there was an improvement in survival with ICIs in the TMB-high white population, this benefit was not seen in Black or Asian patients [159]. Employing ancestry data, a high median TMB value has been shown to be more common among the Black population with NSCLC [157,160]. Thus, a more accurate classification of tumor-only TMB estimation may be achieved by taking into account the genetic ancestry data, especially given the evidence of variation in the germline mutational burden across diverse racial groups [161,162]. It is possible that intracellular factors exhibit variations influencing the response to ICIs across diverse racial groups [163,164]. There is a need for the development of race/ethnicity-based algorithms for tissue-agnostic TMB estimation.

Finally, improvements in clinical trial design for immunotherapy drugs, such as those favoring landmark analysis endpoints, adoptive designs, the integration of immune biomarkers, and improved biostatistical analyses, continue to improve the overall assessment of biomarkers in clinical trials [165]. Incorporating comprehensive pharmacodynamic assessments into clinical trials, which involve the sequencing of proteins, transcriptomics, and the study of molecular pathways and mechanisms, may be integrated into the evaluation of TMB to understand the function of molecular alterations.

## 6. Conclusions

In conclusion, while not a perfect biomarker, with advancements in TMB measurement, standardization initiatives, enhanced testing protocols, TMB characterization in diverse cancer types, its amalgamation with other biomarkers of response to ICIs, dynamic monitoring through bTMB, and the rigorous validation of improved testing methodologies, among various other factors, TMB has the potential for enhanced practical utility in the real-world clinical setting.

## Figures and Tables

**Table 1:** (**A**,**B**) Technical challenges to robustness of Tumor Mutational Burden (TMB) measurement and clinical interpretation.

**(A) Challenges associated with TMB assessment**	
**Tumor/Biopsy Specific**	Cold vs. hot, tumor purity, intra- and inter-tumor heterogeneity, type of biopsy and tumor tissue (fresh versus archival, FFPE, ctDNA), tumor cell quantification, tumor enrichment
**Assay Specific**	Composition, platform, coverage, depth, mutation calling, Input, Indexes, Sensitivity, Specificity, sample and library preparation and conversion, sequencing, exon coverage, data analysis
**Bioinformatics**	Variation in bioinformatic platforms and TMB (germline variants, deduction of driver mutations, CH, resistance genes), VAF, variant calling, Cutoff validation, QC
**Interpretation of results**	Variations in TMB thresholds, variation in patient populations including race/assays used/cancer treatments in different clinical trials, turnaround time, scoring failure rates, intra-patient factors
**(B) Potential solutions**	
**Factors affecting TMB measurement**	**Potential Solution**
**Assay Specific**	
Panel Size	Minimum of 1 Mb of coding sequence is required for reliable measurement
Mutations to be counted for measurement of TMB	Identification of true somatic mutations
	Robust identification of mutations through NGS assay (specimen quality, sequencing depth)
Minimum tumor tissue content	Tumor tissue enrichment (FoundationOne®CDx optimum 30% tumor content)
**Bioinformatics Algorithm**	
Somatic mutation identification	Removal of germline mutation using large population genome sequencing databases
	Using algorithms to further identity somatic mutations
	Removal of pathogenic mutations
Removal of germline variants from rare populations	Calibrating the cut-off values for germline identification from population variant databases.
**Interpretation of Results**	
Histology and clinical-context-specific interpretation of TMB values	Using disease-specific stratification of TMB-high patients
Different cut-offs for different treatments and histologies	Universal/histology-specific cut-offs
**References:** [4,12,25,26,27,28,29,30]	

Abbreviations: CH, clonal hematopoiesis; CDx, companion diagnostic; ctDNA, circulating tumor deoxyribonucleic acid; NGS, next-generation sequencing; Mb, megabase of DNA; QC, quality control; TMB, Tumor Mutational Burden; VAF, variant allele frequency; vs, versus.

**Table 2:** Examples of clinical trials using bTMB as exploratory biomarker.

Clinical Trial	Treatment	Phase/Design	Stage of Cancer	TMB Cut-off (mut/Mb)	TMB Assay	Results	Additional Points
POPLAR trial NCT01903993 [122,123]	Atezolizumab vs. Docetaxel	Phase 2 randomized controlled trial	Previously treated, Stage IIIB or IVNSCLC	≥10 to ≥20	bTMB assay compared to tTMB using Foundation-ACT (FACT) (targets 1.1 Mb coding region)	PFS HR 0.68, 0.57, 0.58 at bTMB cut-offs ≥10, ≥16 and ≥20; OS HR 0.59, 0.56, 0.51 at bTMB cut-offs ≥10, ≥16 and ≥20. mOS 12.6 vs. 9.7 mo in Atezolizumab vs. decetaxel (HR 0.76) At bTMB cut-off of ≥16, mPFS 4.2 vs. 2.9 mo and mOS 13 vs. 7.4 mo in atezolizumab vs. docetaxel	Training set; PPA 64%, NPA 88%. Spearman rho 0.64
OAK Trial NCT02008227 [124,125]	Atezolizumab vs. Docetaxel	Phase 3 randomized controlled trial	Previously treated, Stage IIIB or IV NSCLC	≥10 to ≥20	bTMB assay compared to FoundationACT (FACT) (targets 1.1 Mb coding region)	mOS 13.3 vs. 9.8 mo HR = 0.78.	Validation set; significant PFS benefit for bTMB ≥16 (HR 0.65, *p* = 0.013)
BFAST Cohort C NCT03178552 [126]	Atezolizumab vs. Platinum Chemotherapy	Phase 3	First line Stage IIIB–IV NSCLC	≥16	Foundation Medicine bTMB Clinical Trial Assay (targets 1.1 Mb coding region) *	mFPS 4.5 vs. 4.3 mo, mOS 13.3 vs. 10.3 mo in Atezolizumab vs. chemotherapy (not significant)	Concordance: bTMB CTA ≥ 16 equivalent to a F1 CDx value of 13.6 mut/Mb; 10 mutations were equivalent to 8.3 Mut/Mb, PPA of 82.9%, NPA of 91.5%
B-FIRST NCT02848651 [127]	Atezolizumab	Phase 2	First line Stage IIIB–IVB NSCLC	≥16	FoundationOne bTMB Assay (targets 1.1 Mb coding region)	ORR 17% in ITT, mPFS 5 vs. 3.5 mo in bTMB ≥ 16 vs. <16 (HR 0.8, *p* = 0.35), mOS 23.9 vs. 13.4 mo in the bTMB ≥ 16 vs. <16 group (HR = 0.6, *p* = 0.18) not significant	Exploratory analysis showed patients with MSAF < 1% had significantly longer PFS (6.8 vs. 3.6 mo, *p* = 0.047)
MYSTIC NCT02453282 [128,129]	Durvalumab +/− Tremelimumab vs chemotherapy	Phase 3, Randomized controlled trial	First Line metastatic NSCLC	≥10 to ≥20	bTMB GuardantOMNI ctDNA platform; tTMB FoundationOne® CDx Assay (1.1 Mb coding region)	bTMB ≥ 20 mut/Mb associated with improved OS and FPS. mOS 12.6 mo (HR 0.72 for durvalumab vs chemo); HR 0.74 for Durvalumab + trememelimumab vs. durvalumab	Concordance Spearman rho 0.6
NEPTUNE NCT02542293	Durvalumab + Temelimumab	Phase 3, Randomized controlled trial	First line metastatic NSCLC	≥20 Mut/Mb	GuardantOMNI plasma next-generation sequencing platform	For bTMB ≥ 20 mOS 11.7 vs. 9.1 in durvalumab/tremelimumab vs chemo, HR 0.71; mPFS 4.2 vs. 5.1 mo, HR 0.77. Not significant	No correlation between bTMB and PD-L1 status (Spearman’s rho 0.018)
BUDDY trial NCT04059887 [119]	Atezolizumab	Prospective	Relapsed or metastatic NSCLC		CT-ULTRA	No difference in ORR between bTMB low and high divided by median bTMB of 11.5 mut/Mb	-

* Maximum somatic allele frequency (MSAF) ≥ 1% was used for producing reliable TMB measurement. Abbreviations. bTMB (blood-based Tumor Mutational Burden); ctDNA, circulating tumor DNA; F1 CDx, FoundationOne® companion diagnostic assay; HR, hazard ratio; mo, months; mOS, median overall survival; mPFS, median progression-free survival; mut/Mb, mutations per megabase of DNA; NPA, negative percentage agreement; NSCLC, non-small cell lung cancer; ORR, objective response rate; OS, overall survival; PFS, progression-free survival; PPA, positive percentage agreement; tTMB, tumor tissue Tumor Mutational Burden; vs., versus.

## Data Availability

Data are contained within this article.
